# Effects of COVID-19-targeted non-pharmaceutical interventions on pediatric hospital admissions in North Italian hospitals, 2017 to 2022: a quasi-experimental study interrupted time-series analysis

**DOI:** 10.3389/fpubh.2024.1393677

**Published:** 2024-04-18

**Authors:** Giuseppe Maglietta, Matteo Puntoni, Caterina Caminiti, Andrea Pession, Marcello Lanari, Fabio Caramelli, Federico Marchetti, Alessandro De Fanti, Lorenzo Iughetti, Giacomo Biasucci, Agnese Suppiej, Andrea Miceli, Chiara Ghizzi, Gianluca Vergine, Melodie Aricò, Marcello Stella, Susanna Esposito, Emilia-Romagna Paediatric COVID-19 network, Francesca Diodati

**Affiliations:** ^1^Clinical and Epidemiological Research Unit, University Hospital of Parma, Parma, Italy; ^2^Pediatric Clinic, IRCCS Azienda Ospedaliera Universitaria di Bologna, Bologna, Italy; ^3^Pediatric Emergency Unit, IRCCS Azienda Ospedaliera Universitaria di Bologna, Bologna, Italy; ^4^Pediatric Intensive Care Unit, IRCCS Azienda Ospedaliera Universitaria di Bologna, Bologna, Italy; ^5^Pediatrics and Neonatology Unit, Ravenna Hospital, AUSL Romagna, Ravenna, Italy; ^6^Paediatrics Unit, Santa Maria Nuova Hospital, AUSL-IRCCS of Reggio Emilia, Reggio Emilia, Italy; ^7^Pediatrics Unit, Department of Medical and Surgical Sciences of Mothers, Children and Adults, University of Modena and Reggio Emilia, Modena, Italy; ^8^Pediatrics and Neonatology Unit, Guglielmo da Saliceto Hospital, Piacenza, Italy; ^9^Department of Medicine and Surgery, University of Parma, Parma, Italy; ^10^Pediatric Clinic, University of Ferrara, Ferrara, Italy; ^11^Pediatric Unit, Pavullo Hospital, AUSL Modena, Modena, Italy; ^12^Paediatrics Unit, Maggiore Hospital, Bologna, Italy; ^13^Pediatric Clinic, Rimini Hospital, AUSL Romagna, Rimini, Italy; ^14^Pediatric Unit, G.B. Morgagni – L. Pierantoni Hospital, AUSL Romagna, Forlì, Italy; ^15^Pediatric Unit, AUSL Romagna, Cesena, Italy; ^16^Pediatric Clinic, University Hospital of Parma, Parma, Italy; ^17^Department of Medicine and Surgery, University of Parma, Parma, Italy

**Keywords:** COVID-19 epidemiology, non-pharmaceutical intervention (NPI), quasi-experimental design, observational study, Interrupted Time Series (ITS) regression analysis, time series analysis, diseases of the respiratory system, Mental Disorders

## Abstract

**Background:**

The use of Non-Pharmaceutical Interventions (NPIs), such as lockdowns, social distancing and school closures, against the COVID-19 epidemic is debated, particularly for the possible negative effects on vulnerable populations, including children and adolescents. This study therefore aimed to quantify the impact of NPIs on the trend of pediatric hospitalizations during 2 years of pandemic compared to the previous 3 years, also considering two pandemic phases according to the type of adopted NPIs.

**Methods:**

This is a multicenter, quasi-experimental before-after study conducted in 12 hospitals of the Emilia-Romagna Region, Northern Italy, with NPI implementation as the intervention event. The 3 years preceding the beginning of NPI implementation (in March 2020) constituted the pre-pandemic phase. The subsequent 2 years were further subdivided into a school closure phase (up to September 2020) and a subsequent mitigation measures phase with less stringent restrictions. School closure was chosen as delimitation as it particularly concerns young people. Interrupted Time Series (ITS) regression analysis was applied to calculate Hospitalization Rate Ratios (HRR) on the diagnostic categories exhibiting the greatest variation. ITS allows the estimation of changes attributable to an intervention, both in terms of immediate (level change) and sustained (slope change) effects, while accounting for pre-intervention secular trends.

**Results:**

Overall, in the 60 months of the study there were 84,368 cases. Compared to the pre-pandemic years, statistically significant 35 and 19% decreases in hospitalizations were observed during school closure and in the following mitigation measures phase, respectively. The greatest reduction was recorded for “Respiratory Diseases,” whereas the “Mental Disorders” category exhibited a significant increase during mitigation measures. ITS analysis confirms a high reduction of level change during school closure for Respiratory Diseases (HRR 0.19, 95%CI 0.08–0.47) and a similar but smaller significant reduction when mitigation measures were enacted. Level change for Mental Disorders significantly decreased during school closure (HRR 0.50, 95%CI 0.30–0.82) but increased during mitigation measures by 28% (HRR 1.28, 95%CI 0.98–1.69).

**Conclusion:**

Our findings provide information on the impact of COVID-19 NPIs which may inform public health policies in future health crises, plan effective control and preventative interventions and target resources where needed.

## 1 Introduction

The SARS-CoV-2 epidemic has had little medical consequences for children and adolescents, as incidence of severe forms of COVID-19 in the pediatric population was low and symptoms of infection were generally mild (1, 2). However, young people were deeply affected by the restrictive measures imposed globally to reduce transmission, such as quarantine, lockdown, and social distancing, often referred to as Non-Pharmaceutical Interventions (NPIs), which considerably changed their daily lives (3). They were confined at home for long periods, with limited opportunity for learning and reduced peer contact, together with adults who were often anxious or psychologically stressed by the circumstances, which added to their own discomfort (3, 4). School closure, enforced in many countries with different durations, was particularly relevant for these age groups, as school is where children and adolescents spend most of their time, and have opportunity for both social interactions and intellectual stimulation (5).

The debate on the pros and cons of population-wide restrictions enacted during the COVID-19 pandemic is ongoing. On the one hand, data seems to support the positive effects of NPIs (6–10), particularly in terms of control of virus spread and consequent reduction in mortality (10). On the other hand, some authors emphasize a range of “side effects” of NPIs, including economic, educational, and health repercussions, disproportionately affecting more vulnerable populations, including children, with little health benefits (11). To manage future health crises, therefore, it is crucial that these strategies are further assessed to inform future pandemic policy and avoid past mistakes (12).

The timing and intensity of NPIs against COVID-19 all over the world varied greatly according to local situations (7). Italy, starting from the Northern regions, was the first European country to be affected by the pandemic (13), and enacted very aggressive restrictive policies, including one of the longest school closures in the world (14).

The analysis of hospitalization trends can provide valuable insights into the repercussions of different restrictions adopted over time, needed to prepare for future pandemics. In particular, to estimate the effectiveness of population-level health interventions that have been implemented at a clearly defined point in time, Interrupted Time Series (ITS) regression analysis is the recommended method (15). However, the majority of research on this topic is monocentric (16–19), is restricted to specific pediatric age classes or considers all ages including adults (17, 19–26), focuses on specific diagnoses (9, 16, 17, 19, 21, 25, 27), only looks at Emergency Department (ED) visits (16, 17, 19, 21, 23, 26, 28, 29), or addresses the time period immediately following the pandemic onset without evaluating ongoing effects (18, 25, 30).

We therefore aimed to quantify the impact of NPIs adopted to prevent or control COVID-19 transmission on the trend of hospitalizations, in 12 hospitals in the Emilia-Romagna Region, Northern Italy, during the 2 years following the start of the pandemic, compared with the previous 3 years, considering two pandemic phases according to the type of adopted NPIs.

## 2 Materials and methods

### 2.1 Study design and setting

This is a multicenter, quasi-experimental controlled before-after study, conducted to estimate the change in pediatric hospital admissions during the COVID-19 pandemic compared to the previous period. For disease categories exhibiting the greatest variations, we investigated the effect during school closure and in the subsequent phase when schools were re-opened and mitigation measures were implemented.

This study was conducted in the Emilia-Romagna Region, Northern Italy, which has an overall pediatric population (from 0 to 17 years) of 673,818 subjects (year 2020) (31), who were potentially affected by NPIs.

The overall study period covered from March 2017 to February 2022 (60 months), defining the implementation of NPIs as an intervention event.

### 2.2 Intervention

National lockdown in Italy was imposed from March 11 through May 4th, 2020, after which economic and social activities were gradually resumed. Restrictions were relaxed over the summer and then reintroduced gradually to counter the second wave of the pandemic. On November 6th, 2020, the Italian Government enforced a three-tiered restriction system on a regional basis, using periodic risk assessments by the Ministry of Health (32). Italy also enforced one of the longest school closures in the world (14). Educational institutions of any grade were shut down from late February up to September 2020, after which schools were reopened and mitigation measures were kept in place, such as mask wearing and reduced student social contact, as well as mandatory distance learning for at least 75% of the time in high schools (32). On March 31, 2022, the state of emergency ended in Italy.

In this study, the beginning of NPI implementation was used as delimitation, defining the 3 years prior to March 2020 (from March 2017 to February 2020, 36 months) as the pre-COVID19 phase (PC). Since school closure is thought to have had a more direct impact on young people than other NPIs, the subsequent 2 years were further subdivided into a school closure phase (SC), from March 2020 to September 2020 (7 months) and a mitigation measures phase (MM), from October 2020 to February 2022 (17 months).

### 2.3 Participants

We analyzed data from 12 of the 15 (80%) hospitals in the Emilia-Romagna Region, which provided complete data throughout the study duration. These centers had a catchment area of 574,760 minor inhabitants in 2020 (equal to 85% of the Emilia Romagna region), comprising 211/269 (78%) pediatric beds. Included subjects were patients aged between 0 and 17 years, hospitalized in the considered time frame. Healthy new-borns were excluded from the analysis.

### 2.4 Data sources

Study data were anonymously extracted from the electronic hospital discharge forms (eHDFs), contained in the administrative databases of the Emilia-Romagna Regional Health Trust, and included the following: age, sex, dates of admission and discharge, main diagnosis and up to five secondary diagnoses (i.e., any conditions existing at admission or occurring during hospitalization which influence treatment or length of stay). The diagnoses were coded according to the International Classification of Diseases, Ninth Revision, Clinical Modification (ICD-9-CM).

### 2.5 Statistical analysis

As outcome variables, we considered the monthly frequency of hospitalizations, total and for ICD9-CM categories (the first three characters), during the 60 months considered by the study. To identify which major ICD9-CM categories had the greatest impact, the Standardized Hospitalization Rates (SHR) per 100,000 person-year were used, considering as standard the resident population in Europe in 2020 (the intermediate of the 5 years considered in this study) (33) and adjusting for age and sex. For each diagnostic category, we measured how any of the time periods changed with respect to the previous phase (SC vs. PC, MM vs. PC and MM vs. SC), by estimating the Standardized Hospitalization Rate Ratios (SHRR) and their 95% Confidence Intervals (95% CI). To investigate the effect of NPIs, the ICD9-CM categories exhibiting the greatest change were assessed using ITS regression analysis. This segmented approach allows to estimate changes attributable to an intervention, in terms of overall (as time trend), immediate (as changes in level) and sustained (increase or decrease in the slope) effects, while accounting for pre-intervention secular trends. Since ITS regression models were applied to analyze count data through time, over-dispersion parameter was also evaluated and tested by graphical diagnostic plot and overdispersion test. We modeled admissions using a Poisson generalized linear model; in case *p*-value from Chi-square test of “estat gof” STATA function was less than 0.05 the model switched from Poisson to Quasi Poisson by specifying the parameter scale (x2). The seasonality components were also included into the ITS models to estimate recurrence undulatory patterns of admissions. Winter was defined as January/February/March, Spring as April/May/June, Summer as July/August/September and Autumn as October/ November/ December. In all ITS models, the annual population of the considered provinces was used as off-set allowing to estimate the hospitalization rate. Post-hoc sensitivity analyses were conducted to investigate the impact of children aged 0–1 years old on HRR estimates from ITS modeling, since we assumed that a very small proportion of children in this age group attends day-care. This is an important factor since the school closure is one of the main NPIs under study. All statistical analyses were centralized and performed with STATA (StataCorp. 2023. Stata Statistical Software: Release 18. College Station, TX: StataCorp LLC).

## 3 Results

Overall, in the 5 years of the study and in the 12 participating centers, there were 84,368 cases. Case demographics are shown in Table 1 for each of the three phases: PC, SC, and MM. The sample of admissions was made up of 57.0% males, with the predominant age group being between 0 and 1 year (38.0%). As expected, the hospitalization rate decreased considerably when school closure was enforced with respect to the pre-pandemic time period (2,548 vs. 3,915 × 100,000 person-year). Supplementary Table S1 shows the standardized hospitalization rates by type of primary diagnosis, from highest to lowest.

**Table 1:** Demographic and clinical characteristics of the analyzed sample.

	PC (Mar 1, 2017- Feb 28, 2020) *n* = 56,449		SC (Mar 1, 2020- Sep 30, 2020) *n* = 7,003		MM (Oct 1, 2020- Feb 28, 2022) *n* = 20,916		Whole period (Mar 1, 2017- Feb 28, 2022) *n* = 84,368	
Sex, *n (%) males*	32,262	(57.2)	3,915	(55.9)	11,883	(56.8)	48,060	(57.0)
Age class, *y n (%)*								
0–1	21,285	(37.7)	2,800	(40.0)	7,940	(40.0)	32,025	(38.0)
2–5	14,135	(25.0)	1,383	(19.8)	4,334	(20.7)	19,852	(23.5)
6–11	9,500	(16.8)	1,228	(17.5)	3,406	(16.3)	14,134	(16.8)
12–17	11,529	(20.4)	1,592	(22.7)	5,236	(25.0)	18,357	(21.8)
Secondary diagnoses, *n (%)*								
1st	27,043	(47.9)	3,687	(52.6)	10,447	(50.0)	41,177	(48.8)
2nd	10,723	(19.0)	1,616	(23.1)	4,336	(20.7)	16,675	(19.8)
3rd	4,468	(7.9)	751	(10.7)	1,797	(8.6)	7,016	(8.3)
4th	2,153	(3.8)	361	(5.2)	854	(4.1)	3,368	(4.0)
5th	994	(1.8)	168	(2.4)	378	(1.8)	1,540	(1.8)
Hospitalization annual rate, (95%CI)*	3,915.4 (3,862.4–3,968.5)		2,547.7 (2,504.0–2,591.5)		3,191.0 (3,141.8–3240.1)		3,609.1 (3,557.4–3,660.8)	

PC, pre-COVID19; SC, School closure; MM, Mitigation measures. * Standardized × 100,000 (pop EU 2020).

**Figure 1:**
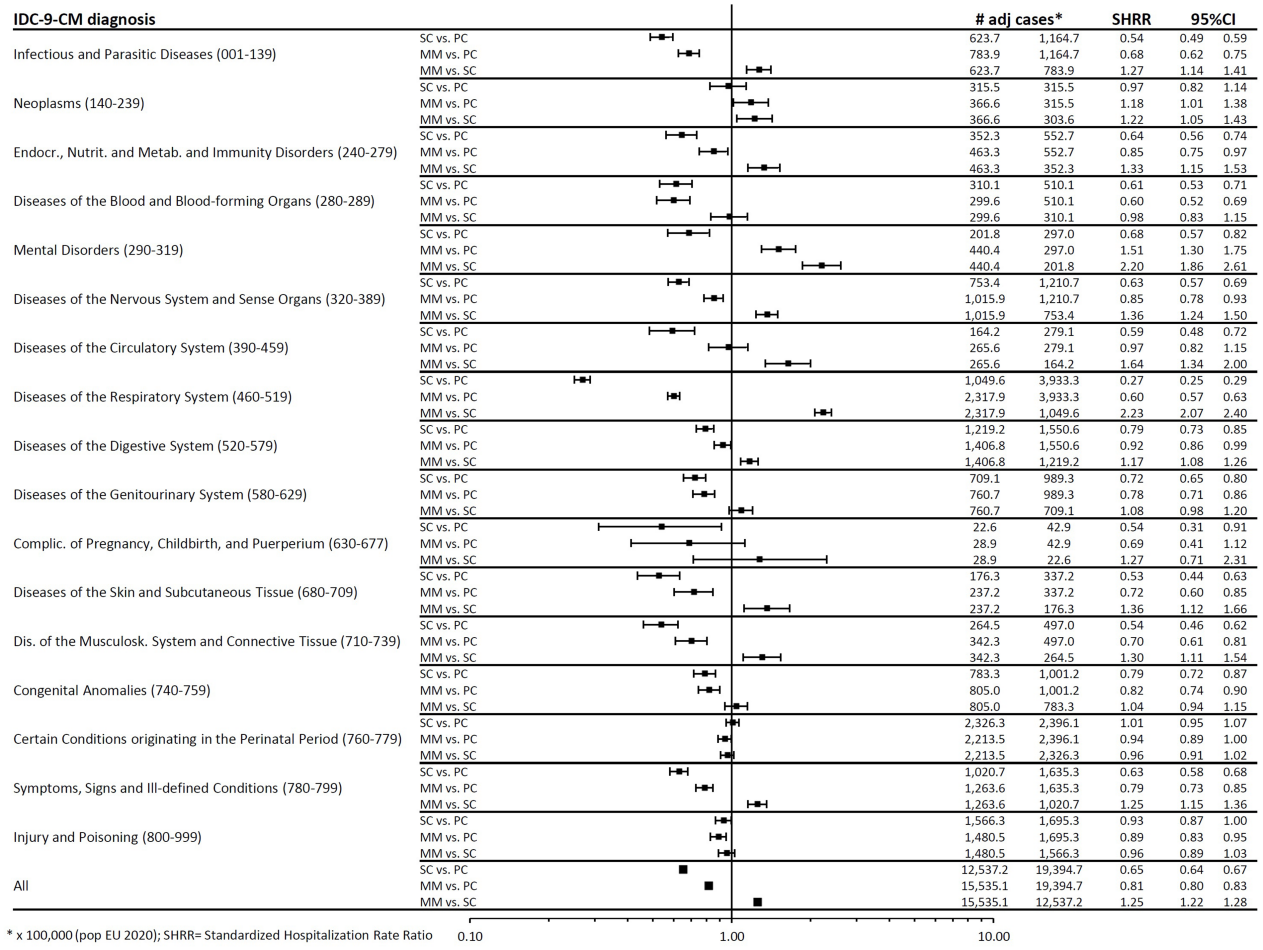
Forest plot of Standardized Hospitalization Rate Ratios (SHRR). Estimates are reported as x 100,000 person-year and are age & sex standardized using as Standard the European resident population in 2020. SHR, standardized hospitalization rate.

Figure 1 shows the comparisons in terms of SHRR, overall and for individual ICD9-CM categories, between SC, MM and PC. Overall, a statistically significant decrease in hospitalizations with respect to pre-pandemic rates was observed both in SC (−35%, SHRR 0.65, 95%CI: 0.64–0.67) and MM (−19%, SHRR 0.81, 0.80–0.83), while a 25% increase (SHRR 1.25, 1.22–1.28) was recorded in MM with respect to SC.

Considering individual ICD-9-CM diagnoses, a generalized reduction was detected during SC for all categories. The greatest reduction (−73%, SHRR 0.27, 95%CI 0.25–0.29) occurred in the “Respiratory Diseases” category, which exhibited the highest frequency of hospitalizations (approximately 4,000 cases/year in the 3 years before the pandemic). In MM, the reduction compared to PC persisted, although less prominent. Only the Mental Disorders category showed a large increase (51%, SHRR 1.51, 95%CI 1.30–1.75).

To measure NPI effects, ITS regression analysis was carried out on overall hospital admissions and on the two categories which stood out for the greatest variation (Respiratory Diseases and Mental Disorders).

Results of the ITS analysis are presented in the following paragraphs.

### 3.1 Any hospitalization

**Figure 2:**
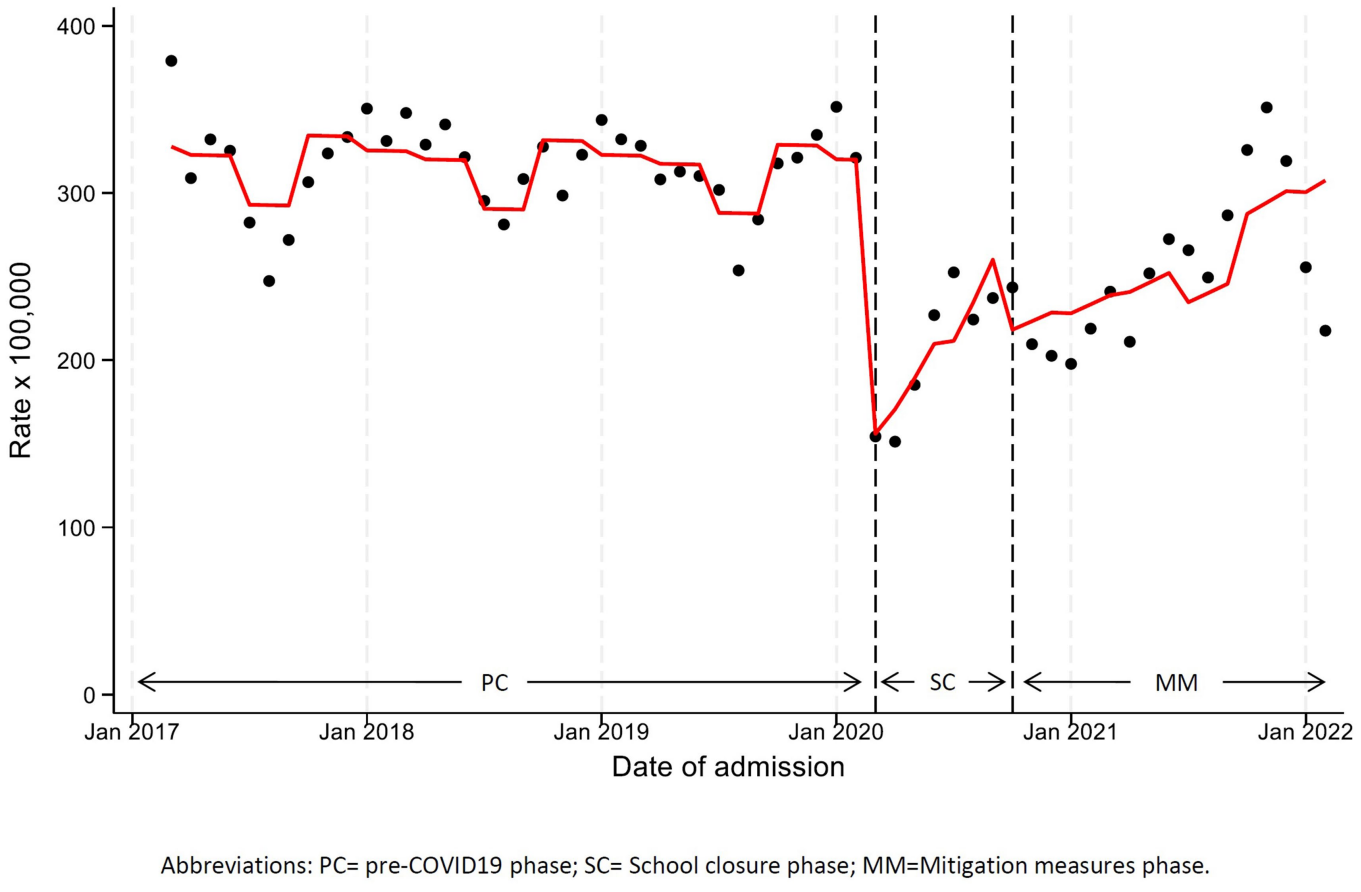
Monthly hospitalization rate any disease with line trend from ITS regression analysis. PC, pre-COVID19 phase; SC, School closure phase; MM, Mitigation measures phase.

As shown in Figure 2 and Table 2, we observed a highly significant decrease in hospitalizations in SC (level change, HRR 0.44, 95%CI 0.35–0.55) and in MM (although of lesser impact, HRR 0.65, 95%CI 0.57–0.75) compared to PC. Unlike the constant hospitalization rate recorded throughout the 3 years before the pandemic, immediately after the collapse of admissions an increasing trend occurred, particularly in SC (slope change, 11% per month, HRR 1.11, 95%CI 1.06–1.16), but also to a lesser extent in MM (slope change, 2% per month, HRR 1.02, 95%CI 1.01–1.03). Hospitalization rates returned to pre-pandemic levels only in autumn 2021 (18 months since the start of the pandemic).

**Table 2:** Interrupted time series analysis results on hospitalizations.

Variable	HRR	95%CI	*p*-value
*Level change^a^*			
SC vs. PC	0.44	0.35–0.55	<0.001
MM vs. PC	0.65	0.57–0.75	<0.001
MM vs. SC	1.48	1.18–1.87	0.001
*Slope change^b^*			
SC vs. PC	1.11	1.06–1.16	<0.001
MM vs. PC	1.02	1.01–1.03	<0.001
MM vs. SC	0.92	0.89–0.95	<0.001
*Time trend^c^*	1.00	0.99–1.01	0.639
*Season*			
Summer	1.00		
Winter	1.12	1.04–1.20	0.003
Spring	1.10	1.02–1.18	0.012
Autumn	1.14	1.06–1.23	<0.001

^a^ Level change refers to an abrupt level change of the Incidence rate between the periods; ^b^ Slope change refers to slope change of the incidence rate over time between the periods; ^c^ Time trend refers to the change of Incidence rate associated with a time unit increase. PC, pre-COVID19 phase; SC, School closure phase; MM, Mitigation measures phase. HRR, Hospitalization Rate Ratio; 95%CI: 95% confidence interval. Pseudo R2 = 0.68. MM vs. SC contrast was manually added for interpretative purposes without *p*-value adjustment for multiple comparison.

### 3.2 Respiratory diseases

**Figure 3:**
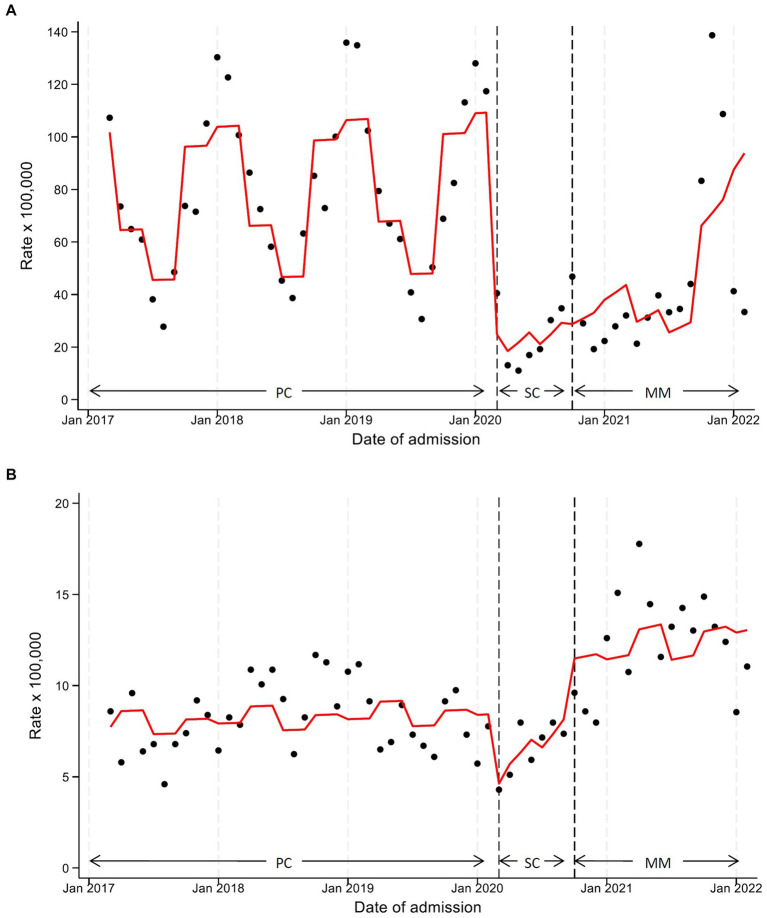
Monthly hospitalization rate for respiratory diseases **(A)** and mental disorders **(B)** with line trend from ITS regression analysis. PC, pre-COVID19 phase; SC, School closure phase; MM, Mitigation measures phase.

The most frequent types of respiratory diseases as primary diagnosis are shown in Supplementary Table S2. This category, which contributed the most to the hospitalization decline, exhibited in SC a statistically significant reduction of 81% in the number of admissions in terms of level change (HRR 0.19, 95%CI 0.08–0.47), and a increase of the monthly slope change of 17% (HRR 1.17, 95%CI 0.97–1.42). A similar but less pronounced decrease was seen during MM, with a statistically significant reduction in terms of level change (HRR 0.26, 95%CI 0.16–0.41), and a 7% increase of the monthly slope change (HRR 1.07, 95%CI 1.03–1.11). The seasonality component analysis showed statistically significant increases from autumn to spring compared to summer (Table 3 and Figure 3A).

**Table 3:** Interrupted time series analysis results on hospitalizations for Respiratory Diseases and Mental Disorders categories.

Variable	HRR	95%CI	*p*-value
Respiratory diseases			
*Level change^a^*			
SC vs. PC	0.19	0.08–0.47	<0.001
MM vs. PC	0.26	0.16–0.41	<0.001
MM vs. SC	1.34	0.52–3.51	0.546
*Slope change^b^*			
SC vs. PC	1.17	0.97–1.42	0.099
MM vs. PC	1.07	1.03–1.11	<0.001
MM vs. SC	0.91	0.75–1.11	0.346
*Time trend^c^*	1.00	0.99–1.01	0.675
*Season*			
Summer	1.00		
Winter	2.25	1.74–2.90	<0.001
Spring	1.42	1.08–1.88	0.012
Autumn	2.10	1.63–2.72	<0.001
Mental disorders			
*Level change^a^*			
SC vs. PC	0.50	0.30–0.82	0.006
MM vs. PC	1.28	0.98–1.69	0.071
MM vs. SC	2.59	1.55–4.34	<0.001
*Slope change^b^*			
SC vs. PC	1.11	1.00–1.23	0.052
MM vs. PC	1.01	0.99–1.03	0.449
MM vs. SC	0.91	0.82–1.01	0.076
*Time trend^c^*	1.00	0.99–1.01	0.541
*Season*			
Summer	1.00		
Winter	1.06	0.90–1.26	0.474
Spring	1.18	1.00–1.40	0.053
Autumn	1.10	0.93–1.30	0.252

^a^ Level change refers to an abrupt level change of the Incidence rate between the periods; ^b^ Slope change refers to slope change of the incidence rate over time between the periods; ^c^ Time trend refers to the change of Incidence rate associated with a time unit increase. PC, Pre-COVID19 phase; SC, School Closure phase; MM, Mitigation Measures phase. HRR, Hospitalization Rate Ratio; 95%CI: 95% confidence interval. Pseudo R2 for respiratory diseases model = 0.69, for mental disorders model = 0.23. MM vs. SC contrasts were manually added for interpretative purposes without p-value adjustment for multiple comparison.

### 3.3 Mental disorders

As evident in Figure 3B, although hospitalizations in this category underwent a substantial decrease at the start of SC, we observed a sharp increasing trend until MM, when hospitalization rates exceeded pre-pandemic levels. ITS analysis (Table 3) detected in SC a statistically significant 50% reduction in level change (HRR 0.50, 95%CI 0.30–0.82) and a borderline statistically significant 11% increase in the monthly slope change (HRR 1.11, 95%CI 1.00–1.23), compared to PC. Comparing MM with the pre-pandemic situation, a 28% level change increase was observed (HRR 1.28, 95%CI 0.98–1.69), while the monthly slope change remained unchanged (HRR 1.01, 95%CI 0.99–1.03). Finally, comparing MM vs. SC, we recorded a strong increase in the level change of about 2.6 times (HRR 2.59, 95%CI 1.55–4.34). The seasonality component analysis showed statistically significant increases of admission during spring (HRR 1.18, 95%CI 1.00–1.40) versus summer (Table 3 and Figure 3B).

### 3.4 Subgroup analysis by sex and age

We performed subgroup analyses considering gender and age categories. Gender differences were not found for Respiratory Diseases, whereas for Mental Disorders the increase in MM vs. PC seemed to be significantly stronger in females vs. males (MM vs. PC: 1.66, 1.19–2.33 vs. 1.28, 0.98–1.69, respectively) (Supplementary Figures S1, S2 and Supplementary Tables S4, S5). Although incidence rates of Respiratory Diseases differed between ages 0–5 and 12–17, HRR estimates did not exhibit relevant differences (Supplementary Figures S3, S4 and Supplementary Tables S6, S7). Concerning Mental Disorders, in the 12–17 age subgroup, the HRR of level change in MM increased from a non-statistically significant 1.28 (*p* = 0.071) to a highly statistically significant 1.66 (*p* < 0.001), even though the slope change was almost absent and identical (HRR 1.01) (Supplementary Figure S5 and Supplementary Table S8).

### 3.5 Sensitivity analyses

Supplementary Tables S9, S10 display the results of sensitivity analyses performed excluding children aged 0–1 years old, who represent about 38% of overall hospital admissions. Considering both admissions for any cause and for the Respiratory Diseases and Mental Disorders categories, sensitivity analyses did not reveal differences sufficient to suggest that the proportion of children aged 0 to 1 year significantly skewed the results of our analyses.

## 4 Discussion

This is the first European study on the impact of COVID-19 NPIs on the trend of pediatric hospitalizations conducted in a wide area severely hit by the pandemic, covering an extended pandemic period (24 months). The use of appropriate analysis through ITS regression makes our findings and corresponding conclusions reliable. In fact, existing research on the effects of NPIs mostly consists of modeling studies, implying a lack of empirical, real-world data, or uses descriptive statistics on admission trends (34).

Overall, our results showed that the number of pediatric hospital admissions dropped by more than 50% in the first months of the lockdown period, and then began to rise, achieving pre-pandemic hospitalization levels only 2 years later. This considerable, long-lasting reduction appears to be mainly determined by a decrease in the occurrence of infectious diseases (the most frequent cause for hospitalization in children), mainly affecting the respiratory system. However, these results may also be due to a change in health-seeking behaviors of parents, who might have chosen not to attend hospital with their sick children for fear of contagion (9, 35). Moreover, the decrease may be attributed to a tendency to avoid hospitalizing children with minor health problems. Supporting this hypothesis is the fact that admissions for childhood neoplasms remained constant, suggesting that healthcare services were maintained for severe illnesses. A similar observation was made by Wang et al. (9), who found a 55% reduction in admissions for all-cause respiratory diseases, in line with our finding, and a smaller reduction in admissions for childhood neoplasms.

Interesting results emerged from ITS analyses conducted on the two disease categories exhibiting the largest variation, which recorded opposite trends. For Respiratory Diseases, we observed a marked reduction of hospitalizations which persisted throughout school closure and for the most part of the subsequent time period when less stringent mitigation measures were enforced, in the absence of typical seasonal epidemic peaks. Conversely, for Mental Disorders an immediate decline of admissions was detected in the first 2 months of lockdown, followed by an incremental trend, on average by 11% monthly. These trends need to be further investigated using hospitalization data recorded in the following years, to understand whether the effects persist, or whether at the end of the pandemic hospitalizations return to pre-pandemic levels.

Some plausible reasons for these results exist. Regarding Respiratory Diseases, the drop in admissions is likely to be related to the impact of mask-wearing, hand washing, and social distancing on the interruption of person-to-person viral or bacterial transmission, as also discussed by Wang et al. (9). The reduction may also be partly due to a “virus interference phenomenon” among respiratory viruses, whereby the infection of one virus can partially prevent or inhibit the infection of another virus in the same host (36). The contribution of this factor is however likely to be marginal compared to the absence of influenza epidemics and other respiratory infections following social distancing, which has been reported and commented in the literature (37, 38). Concerning mental health, the negative effects may have taken longer to manifest, but once developed they may not resolve easily even if restrictions are lifted, instead requiring much time and specific care to be removed (3).

The results of this study can contribute to the current debate on benefits and harms of individual NPIs, which is not a simple one, also because it is hard to separate the impact of one measure from that of other interventions introduced simultaneously. Concerning the pediatric population in particular, it would be essential to elucidate the role of school closures on the control of pandemic spread (39). Recent reviews (12, 34, 40) suggested that measures implemented in the school setting may have limited the number or proportion of cases and deaths among adults, and delayed the progression of the pandemic. This seems to contrast with a report on data from Sweden, where school closure was only reserved for upper secondary schools, indicating that the number of deaths per population unit was lower than most other high-income countries that applied stringent school closure policies (41). On the other hand, the literature also highlights negative consequences of these measures on children’s health and education. As reported by UNICEF (42), school closures disrupted the provision of educational (and in some cases health and nutritional) services, increased emotional distress and mental health problems, an prevented access to a wide range of school-provided services, including school meals, monitoring of health and welfare, social skills training, and services targeted to children with special needs. Furthermore, as schools moved online, impoverished children experienced dramatic educational setbacks contributing to inequalities and long-term hardship (42).

Within the current debate, our findings also highlight that evaluating the trade-offs between positive and negative consequences of NPI implementation during pandemics is a complex task. In particular, as commented above, the decrease in hospitalizations for Respiratory Diseases after the beginning of the outbreak may be due both to the hesitancy in attending hospitals, certainly an undesired effect, and to the reduction of respiratory infections due to lockdown measures, a welcome benefit.

One of the main strengths of this research lies in the use of ITS analysis, one of the strongest evaluative designs when randomization is not possible (15). Furthermore, the study involves numerous hospitals, which makes results robust and increases their generalizability. Also, analyzed data concern the first European area hit by the pandemic, where aggressive restrictive measures were immediately adopted since the start of the outbreak and maintained for an extended period, are restricted to one endpoint (pediatric hospitalizations) and include COVID and non-COVID hospitalizations. Finally, the study covers a wide timeframe, longer than most similar research, which enabled to verify the impact of NPIs in the long-term.

This study has some limitations. Firstly, data were taken from hospital administrative databases and were not collected prospectively for this research. However, the data quality is supposed to be similar in the years we compared; thus, this aspect should not impact interpretation. Secondly, we did not attempt to discriminate between new versus recurrent hospitalizations. Such discrimination would be important to understand whether the observed changes were due to the onset of a new condition or to the exacerbation of existing problems. Thirdly, since the analysis used data collected retrospectively without formal power analysis, we cannot exclude the risk of false negative findings in the case of low-prevalence diagnoses. Lastly, we did not attempt to investigate the potential role of different waves of variants of the SARS-CoV-2 virus which were predominant in the 2 years covered by the study, because it was not an objective of our research. This may have led to an overestimation of the effect of NPIs on hospital admissions.

## 5 Conclusion

The results of this and other studies on the impact of COVID-19 NPIs on children provide information needed to guide and target interventions in the event of future pandemics, and to plan the allocation of resources where they are needed most. However, the different plausible interpretations of our findings make it difficult to inform about the trade-offs between benefits and negative consequences of NPI strategies during pandemics. Rigorous research should be conducted to understand whether the reduction in pediatric hospital admissions we observed over a two-year period has affected child and adolescent health. Meta-analyses are needed to quantify the contribution to observed effects of individual mitigation actions, to better determine the appropriateness of their introduction, timing and intensity.

## Data availability statement

The data analyzed in this study is subject to the following licenses/restrictions: the raw data supporting the conclusions of this article will be made available by the authors upon a motivated request to the corresponding author. Requests to access these datasets should be directed to ccaminiti@ao.pr.it.

## Ethics statement

The studies involving humans were approved by AVEN (Area Vasta Emilia Nord) Ethics Committee. The studies were conducted in accordance with the local legislation and institutional requirements. The ethics committee/institutional review board waived the requirement of written informed consent for participation from the participants or the participants’ legal guardians/next of kin because waiver for informed consent was obtained from the Italian Data Protection Authority (Garante della Privacy), because of feasibility issues (more than 80,000 subjects should have been contacted).

## Group members of Emilia-Romagna Paediatric COVID-19 network

Collective authors who fulfill all four ICMJE authorship criteria.

Francesca Diodati, Chiara Maria Palo: Clinical and Epidemiological Research Unit, University Hospital of Parma, Parma, Italy; Angela Miniaci, Luca Bertelli: Pediatric Clinic, IRCCS Azienda Ospedaliera Universitaria di Bologna, Bologna, Italy; Giovanni Biserni: Pediatric Emergency Unit, IRCCS Azienda Ospedaliera Universitaria di Bologna, Bologna, Italy; Angela Troisi, Alessandra Iacono: Pediatrics and Neonatology Unit, Ravenna Hospital, AUSL Romagna, Ravenna, Italy; Federico Bonvicini, Domenico Bartolomeo, Andrea Trombetta: Paediatrics Unit, Santa Maria Nuova Hospital, AUSL-IRCCS of Reggio Emilia, Reggio Emilia, Italy; Tommaso Zini: Pediatrics Unit, Department of Medical and Surgical Sciences of Mothers, Children and Adults, University of Modena and Reggio Emilia, Modena, Italy; Nicoletta de Paulis: Pediatrics and Neonatology Unit, Guglielmo da Saliceto Hospital, Piacenza, Italy; Cristina Forest: Pediatric Clinic, University of Ferrara, Ferrara, Italy; Battista Guidi: Pediatric Unit, Pavullo Hospital, AUSL Modena, Pavullo, Italy; Francesca Di Florio: Paediatrics Unit, Maggiore Hospital, Bologna, Italy; Enrico Valletta, Francesco Accomando: Pediatric Unit, G.B. Morgagni - L. Pierantoni Hospital, AUSL Romagna, Forlì; Greta Ramundo, Alberto Argentiero, Valentina Fainardi, Michela Deolmi: Pediatric Clinic, University Hospital, Department of Medicine and Surgery, University of Parma, Parma, Italy.

## Author contributions

GM: Formal analysis, Writing – original draft, Writing – review & editing, Data curation, Methodology. MP: Data curation, Formal analysis, Methodology, Writing – original draft, Writing – review & editing. CC: Formal analysis, Writing – original draft, Writing – review & editing, Conceptualization, Validation. AP: Investigation, Resources, Resources, Writing – review & editing. ML: Investigation, Resources, Writing – review & editing. FC: Investigation, Resources, Writing – review & editing. FM: Investigation, Resources, Writing – review & editing. AF: Investigation, Resources, Writing – review & editing. LI: Investigation, Resources, Writing – review & editing. GB: Investigation, Resources, Writing – review & editing. AS: Investigation, Resources, Writing – review & editing. AM: Investigation, Resources, Writing – review & editing. CG: Investigation, Resources, Writing – review & editing. GV: Investigation, Resources, Writing – review & editing. MA: Investigation, Resources, Writing – review & editing. MS: Investigation, Resources, Writing – review & editing. SE: Conceptualization, Investigation, Project administration, Supervision, Writing – review & editing.
