# Erratum: Effects of COVID-19-targeted non-pharmaceutical interventions on pediatric hospital admissions in North Italian hospitals, 2017 to 2022: a quasi-experimental study interrupted time-series analysis

**DOI:** 10.3389/fpubh.2024.1437966

**Published:** 2024-06-18

**Authors:** Frontiers Production Office

**Affiliations:** Frontiers Media SA, Lausanne, Switzerland

**Keywords:** COVID-19 epidemiology, non-pharmaceutical intervention (NPI), quasi-experimental design, observational study, Interrupted Time Series (ITS) regression analysis, time series analysis, diseases of the respiratory system, Mental Disorders

Due to a production error, the consortium was incorrectly listed in the article. The author list, citation, and copyright should appear as:

Maglietta G, Puntoni M, Caminiti C, Pession A, Lanari M, Caramelli F, Marchetti F, De Fanti A, Iughetti L, Biasucci G, Suppiej A, Miceli A, Ghizzi C, Vergine G, Aricò M, Stella M, Esposito S and Emilia-Romagna Paediatric COVID-19 network

The heading “Collaborators” in the article has been edited to “Group members of Emilia-Romagna Paediatric COVID-19 network” accordingly.

The publisher apologizes for this mistake. The original article has been updated.

