# Supplementary material for: Effects of COVID-19-targeted non-pharmaceutical interventions on pediatric hospital admissions in North Italian hospitals, 2017 to 2022: a quasi-experimental study interrupted time-series analysis
Source: Front Public Health. 2024 Apr 18;12:1393677. doi: 10.3389/fpubh.2024.1393677 (PMC11064846; doi:10.3389/fpubh.2024.1393677)
Supplement: Supplementary file 1 [file Data_Sheet_1.docx]

**Supplementary tables**

Supplementary Table S1: Annual Standardized Hospitalization Rates x 100,000 (pop EU 2020) by type of primary diagnosis, from the highest to the lowest.

Supplementary Table S2: Type of respiratory diseases on primary diagnosis, sorted by frequency (>1% on total, N=15,057).

Supplementary Table S3: Type of mental disorders on primary diagnosis, sorted by frequency (>1% on total, N=1,650).

Supplementary Table S4: Interrupted time series analysis results for respiratory diseases in females.

Supplementary Table S5: Interrupted time series analysis results for mental disorders in females.

Supplementary Table S6: Interrupted time series analysis results for respiratory diseases in children aged 0-5y.

Supplementary Table S7: Interrupted time series analysis results for respiratory diseases in adolescents (age 12-17).

Supplementary Table S8: Interrupted time series analysis results for mental disorders in adolescents (age 12-17).

Supplementary Table S9: Interrupted time series analysis results on hospitalisations, excluding 0-1 years old subjects (sensitivity analysis).

Supplementary Table S10: Interrupted time series analysis results on hospitalisations for Respiratory Diseases and Mental Disorders categories, excluding 0-1years old subjects (sensitivity analysis).

| Supplementary Table S1: Annual Standardized Hospitalization Rates (95%CI) x 100,000 (pop EU 2020) by type of primary diagnosis, from the highest to the lowest. | | | | |
| --- | --- | --- | --- | --- |
| Diagnosis (ICD9-CM code) | PC phase  (Mar 1, 2017-  Feb 28, 2020)  n=56,449 | SC phase  (Mar 1, 2020-  Sep 30, 2020)  n=7,003 | MM phase  (Oct 1, 2020-  Feb 28, 2022)  n=20,916 |  |
| Respiratory System (460-519) | 794.1  (769.1-819.1) | 213.3  (200.2-226.4) | 476.1  (456.1-496.1) |  |
| Certain Conditions originating in the Perinatal Period (760-779) | 483.7  (464.5-502.9) | 472.7  (453.3-492.2) | 454.7  (435.2-474.1) |  |
| Injury and Poisoning (800-999) | 342.3  (326-358.6) | 318.3  (302.5-334.1) | 304.1  (288.6-319.6) |  |
| Digestive System (520-579) | 313  (297.4-328.6) | 247.8  (233.8-261.7) | 289  (273.8-304.1) |  |
| Symptoms, Signs and Ill-defined Conditions (780-799) | 330.1  (313.9-346.3) | 207.4  (194.4-220.4) | 259.5  (244.9-274.2) |  |
| Nervous System and Sense Organs (320-389) | 244.4  (230.6-258.3) | 153.1  (142.1-164.2) | 208.7  (195.7-221.7) |  |
| Infectious and Parasitic Diseases (001-139) | 235.1  (221.4-248.9) | 126.8  (116.6-136.9) | 161  (149.3-172.7) |  |
| Congenital Anomalies (740-759) | 202.1  (189.4-214.9) | 159.2  (147.7-170.7) | 165.4  (153.6-177.2) |  |
| Genitourinary System (580-629) | 199.7  (187.1-212.3) | 144.1  (133.3-154.9) | 156.2  (144.9-167.6) |  |
| Endocrine, Nutrition and Metabolism and Immunity Disorders (240-279) | 111.6  (102.1-121) | 71.6  (64-79.2) | 95.2  (86.3-104) |  |
| Blood and Blood-forming Organs (280-289) | 103  (94-112) | 63  (55.9-70.1) | 61.5  (54.4-68.6) |  |
| Musculoskeletal System and Connective Tissue (710-739) | 100.3  (91.5-109.1) | 53.8  (47.3-60.3) | 70.3  (62.9-77.7) |  |
| Mental Disorders (290-319) | 60  (53.1-66.8) | 41  (35.4-46.6) | 90.5  (82.2-98.8) |  |
| Neoplasms (140-239) | 63.7  (56.6-70.8) | 61.7  (54.7-68.7) | 75.3  (67.5-83.1) |  |
| Skin and Subcutaneous Tissue (680-709) | 68.1  (60.7-75.4) | 35.8  (30.5-41.2) | 48.7  (42.4-55) |  |
| Circulatory System (390-459) | 56.3  (49.7-63) | 33.4  (28.2-38.5) | 54.5  (47.9-61.2) |  |
| Complications of Pregnancy, Childbirth, and Puerperium (630-677) | 8.7  (6.1-11.3) | 4.6  (2.7-6.5) | 5.9  (3.8-8.1) |  |
| Abbreviations: PC= pre-COVID19; SC= School closure; MM=Mitigation measures. | | | |  |

Supplementary Table S2: Type of respiratory diseases on primary diagnosis, sorted by frequency (>1% on total, N=15,057).

|  | N. | % |
| --- | --- | --- |
| Hypertrophy of tonsil with adenoids (474.10) | 2,298 | 15.3% |
| Acute Respiratory failure (518.81) | 1,808 | 12.0% |
| Acute bronchiolitis due to respiratory syncytial virus (RSV) (466.11) | 1,579 | 10.5% |
| Acute bronciolitis due to other infectious organisms (466.19) | 746 | 5.0% |
| Hypertrophy of adenoids alone (474.12) | 694 | 4.6% |
| Bronchopneumonia organism unspecified (485) | 611 | 4.1% |
| Chronic tonsillitis and adenoiditis (474.02) | 568 | 3.8% |
| Acute bronchitis (466.0) | 551 | 3.7% |
| Chronic tonsillitis (474.00) | 522 | 3.5% |
| Bacterial pneumonia unspecified (482.9) | 382 | 2.5% |
| Acute bronchospasm (519.11) | 364 | 2.4% |
| Acute upper respiratory infections of unspecified site (465.9) | 310 | 2.1% |
| Pneumonia organism unspecified (486) | 310 | 2.1% |
| Acute upper respiratory infections of other multiple sites (465.8) | 291 | 1.9% |
| Acute tonsillitis (463) | 272 | 1.8% |
| Acute pharyngitis (462) | 265 | 1.8% |
| Chronic adenoiditis (474.01) | 230 | 1.5% |
| Adenoid vegetations (474.2) | 179 | 1.2% |
| Influenza with other respiratory manifestations (487.1) | 177 | 1.2% |
| Influenza with other manifestations (487.8) | 174 | 1.2% |
| Pneumonia due to respiratory syncytial virus (480.1) | 162 | 1.1% |

Supplementary Table S3: Type of mental disorders on primary diagnosis, sorted by frequency (>1% on total, N=1,650).

|  | N. | % |
| --- | --- | --- |
| Anorexia nervosa (307.1) | 270 | 16.4% |
| Eating disorder unspecified (307.50) | 124 | 7.5% |
| Excitative type psychosis (298.1) | 50 | 3.0% |
| Conversion disorder (300.11) | 48 | 2.9% |
| Tension headache (307.81) | 41 | 2.5% |
| Adjustment disorder with mixed disburbance of emotions and conduct (309.4) | 40 | 2.4% |
| Anxiety state unspecified (300.00) | 36 | 2.2% |
| Unspecified psychosis (298.9) | 34 | 2.1% |
| Adjustment disorder with mixed anxiety and depressed mood (309.28) | 33 | 2.0% |
| Unspecified delay in development (315.9) | 29 | 1.8% |
| Autistic disorder, current or active state (299.00) | 28 | 1.7% |
| Predominant psychomotor disturbance (308.2) | 28 | 1.7% |
| Undifferentiated somatoform disorder (300.82) | 23 | 1.4% |
| Borderline personality disorder (301.83) | 23 | 1.4% |
| Other specified episodic mood disorder (296.99) | 22 | 1.3% |
| Generalized anxiety disorder (300.02) | 22 | 1.3% |
| Other and unspecified special symptoms or syndromes not elsewhere classified (307.9) | 22 | 1.3% |
| Mixed disturbance of conduct and emotions (312.4) | 21 | 1.8% |
| Unspecified nonpsychotic mental disorder (300.9) | 20 | 1.2% |
| Unspecified personality disorder (301.9) | 20 | 1.2% |
| Oppositional defiant disorder (313.81) | 18 | 1.1% |
| Other disorders of eating (307.59) | 17 | 1.0% |
| Unspecified disturbance of conduct (312.9) | 17 | 1.0% |

Supplementary Table S4: Interrupted time series analysis results for respiratory diseases in females.

| **Variable** | **HRR** | **95%CI** | **p-Value** |
| --- | --- | --- | --- |
|  |  |  |  |
| *Level change^a^* |  |  |  |
| SC vs. PC | 0.22 | 0.09-0.56 | 0.001 |
| MM vs. PC | 0.24 | 0.14-0.40 | <0.001 |
| MM vs. SC | 1.08 | 0.40-2.93 | 0.886 |
|  |  |  |  |
| *Slope change^b^* |  |  |  |
| SC vs. PC | 1.14 | 0.93-1.40 | 0.193 |
| MM vs. PC | 1.08 | 1.04-1.12 | <0.001 |
| MM vs. SC | 0.94 | 0.77-1.16 | 0.577 |
|  |  |  |  |
| *Time trend^c^* | 1.00 | 0.99-1.01 | 0.662 |
|  |  |  |  |
| *Season* |  |  |  |
| Summer | 1.00 |  |  |
| Winter | 2.25 | 1.71-2.95 | <0.001 |
| Spring | 1.40 | 1.04-1.89 | 0.026 |
| Autumn | 2.11 | 1.60-2.77 | <0.001 |
|  |  |  |  |
| ^a^ Level change refers to an abrupt level change of the Incidence rate between the periods; ^b^ Slope change refers to slope change of the incidence rate over time between the periods. ^c^Time trend refers to the change of Incidence rate associated with a time unit increase. PC= pre-COVID19 phase; SC= School closure phase; MM=Mitigation measures phase. Abbreviations: HRR= Hospitalization Rate Ratio; 95%CI: 95% confidence interval. Note: MM vs. SC contrast was manually added for interpretative purposes without p-value adjustment for multiple comparison. | | | |

Supplementary Table S5: Interrupted time series analysis results for mental disorders in females.

| **Variable** | **HRR** | **95%CI** | **p-Value** |
| --- | --- | --- | --- |
|  |  |  |  |
| *Level change^a^* |  |  |  |
| SC vs. PC | 0.45 | 0.23-0.89 | 0.022 |
| MM vs. PC | 1.66 | 1.19-2.33 | 0.003 |
| MM vs. SC | 3.69 | 1.86-7.34 | <0.001 |
|  |  |  |  |
| *Slope change^b^* |  |  |  |
| SC vs. PC | 1.13 | 0.98-1.30 | 0.084 |
| MM vs. PC | 1.01 | 0.99-1.04 | 0.402 |
| MM vs. SC | 0.89 | 0.78-1.03 | 0.115 |
|  |  |  |  |
| *Time trend^c^* | 1.00 | 0.99-1.01 | 0.934 |
|  |  |  |  |
| *Season* |  |  |  |
| Summer | 1.00 |  |  |
| Winter | 1.01 | 0.81-1.25 | 0.954 |
| Spring | 1.23 | 1.00-1.52 | 0.050 |
| Autumn | 1.13 | 0.92-1.38 | 0.263 |
|  |  |  |  |
| ^a^ Level change refers to an abrupt level change of the Incidence rate between the periods; ^b^ Slope change refers to slope change of the incidence rate over time between the periods. ^c^Time trend refers to the change of Incidence rate associated with a time unit increase. PC= pre-COVID19 phase; SC= School closure phase; MM=Mitigation measures phase. Abbreviations: HRR= Hospitalization Rate Ratio; 95%CI: 95% confidence interval. Note: MM vs. SC contrast was manually added for interpretative purposes without p-value adjustment for multiple comparison. | | | |

Supplementary Table S6: Interrupted time series analysis results for respiratory diseases in children aged 0-5y.

| **Variable** | **HRR** | **95%CI** | **p-Value** |
| --- | --- | --- | --- |
|  |  |  |  |
| *Level change^a^* |  |  |  |
| SC vs. PC | 0.19 | 0.07-0.56 | 0.002 |
| MM vs. PC | 0.24 | 0.14-0.41 | <0.001 |
| MM vs. SC | 1.23 | 0.39-3.90 | 0.728 |
|  |  |  |  |
| *Slope change^b^* |  |  |  |
| SC vs. PC | 1.17 | 0.92-1.48 | 0.206 |
| MM vs. PC | 1.09 | 1.04-1.13 | <0.001 |
| MM vs. SC | 0.93 | 0.73-1.18 | 0.558 |
|  |  |  |  |
| *Time trend^c^* | 1.00 | 0.99-1.01 | 0.670 |
|  |  |  |  |
| *Season* |  |  |  |
| Summer | 1.00 |  |  |
| Winter | 2.85 | 2.08-3.92 | <0.001 |
| Spring | 1.65 | 1.17-2.33 | 0.004 |
| Autumn | 2.57 | 1.87-3.54 | <0.001 |
|  |  |  |  |
| ^a^ Level change refers to an abrupt level change of the Incidence rate between the periods; ^b^ Slope change refers to slope change of the incidence rate over time between the periods. ^c^Time trend refers to the change of Incidence rate associated with a time unit increase. PC= pre-COVID19 phase; SC= School closure phase; MM=Mitigation measures phase. Abbreviations: HRR= Hospitalization Rate Ratio; 95%CI: 95% confidence interval. Note: MM vs. SC contrast was manually added for interpretative purposes without p-value adjustment for multiple comparison. | | | |

Supplementary Table S7: Interrupted time series analysis results for respiratory diseases in adolescents (age 12-17).

| **Variable** | **HRR** | **95%CI** | **p-Value** |
| --- | --- | --- | --- |
|  |  |  |  |
| *Level change^a^* |  |  |  |
| SC vs. PC | 0.36 | 0.20-0.63 | <0.001 |
| MM vs. PC | 0.43 | 0.31-0.60 | <0.001 |
| MM vs. SC | 1.21 | 0.65-2.25 | 0.549 |
|  |  |  |  |
| *Slope change^b^* |  |  |  |
| SC vs. PC | 1.07 | 0.95-1.21 | 0.245 |
| MM vs. PC | 1.02 | 0.99-1.05 | 0.129 |
| MM vs. SC | 0.95 | 0.85-1.07 | 0.426 |
|  |  |  |  |
| *Time trend^c^* | 1.00 | 0.99-1.01 | 0.229 |
|  |  |  |  |
| *Season* |  |  |  |
| Summer | 1.00 |  |  |
| Winter | 1.08 | 0.90-1.28 | 0.416 |
| Spring | 0.88 | 0.73-1.07 | 0.196 |
| Autumn | 1.09 | 0.91-1.30 | 0.339 |
|  |  |  |  |
| ^a^ Level change refers to an abrupt level change of the Incidence rate between the periods; ^b^ Slope change refers to slope change of the incidence rate over time between the periods. ^c^Time trend refers to the change of Incidence rate associated with a time unit increase. PC= pre-COVID19 phase; SC= School closure phase; MM=Mitigation measures phase. Abbreviations: HRR= Hospitalization Rate Ratio; 95%CI: 95% confidence interval. Note: MM vs. SC contrast was manually added for interpretative purposes without p-value adjustment for multiple comparison. | | | |

Supplementary Table S8: Interrupted time series analysis results for mental disorders, in adolescents (age 12-17).

| **Variable** | **HRR** | **95%CI** | **p-Value** |
| --- | --- | --- | --- |
|  |  |  |  |
| *Level change^a^* |  |  |  |
| SC vs. PC | 0.45 | 0.24-0.83 | 0.010 |
| MM vs. PC | 1.66 | 1.22-2.24 | 0.001 |
| MM vs. SC | 3.68 | 2.00-6.80 | <0.001 |
|  |  |  |  |
| *Slope change^b^* |  |  |  |
| SC vs. PC | 1.14 | 1.01-1.29 | 0.031 |
| MM vs. PC | 1.01 | 0.99-1.03 | 0.436 |
| MM vs. SC | 0.88 | 0.78-1.00 | 0.044 |
|  |  |  |  |
| *Time trend^c^* | 1.00 | 0.99-1.01 | 0.855 |
|  |  |  |  |
| *Season* |  |  |  |
| Summer | 1.00 |  |  |
| Winter | 0.94 | 0.78-1.13 | 0.511 |
| Spring | 1.06 | 0.88-1.28 | 0.555 |
| Autumn | 1.07 | 0.89-1.28 | 0.490 |
|  |  |  |  |
| ^a^ Level change refers to an abrupt level change of the Incidence rate between the periods; ^b^ Slope change refers to slope change of the incidence rate over time between the periods. ^c^Time trend refers to the change of Incidence rate associated with a time unit increase. PC= pre-COVID19 phase; SC= School closure phase; MM=Mitigation measures phase. Abbreviations: HRR= Hospitalization Rate Ratio; 95%CI: 95% confidence interval. Note: MM vs. SC contrast was manually added for interpretative purposes without p-value adjustment for multiple comparison. | | | |

| Supplementary Table S9: Interrupted time series analysis results on hospitalisations, excluding 0-1 years old subjects (sensitivity analysis). | | | |
| --- | --- | --- | --- |
| **Variable** | **HRR** | **95%CI** | **p-Value** |
|  |  |  |  |
| *Level change^a^* |  |  |  |
| SC vs. PC | 0.35 | 0.27-0.44 | <0.001 |
| MM vs. PC | 0.65 | 0.57-0.74 | <0.001 |
| MM vs. SC | 1.87 | 1.45-2.40 | <0.001 |
|  |  |  |  |
| *Slope change^b^* |  |  |  |
| SC vs. PC | 1.14 | 1.09-1.20 | <0.001 |
| MM vs. PC | 1.02 | 1.01-1.03 | <0.001 |
| MM vs. SC | 0.89 | 0.85-0.94 | <0.001 |
|  |  |  |  |
| *Time trend^c^* | 1.00 | 0.99-1.01 | 0.559 |
|  |  |  |  |
| *Season** |  |  |  |
| Summer | 1.00 |  |  |
| Winter | 1.02 | 0.95-1.10 | 0.550 |
| Spring | 1.11 | 1.03-1.20 | 0.005 |
| Autumn | 1.06 | 0.99-1.15 | 0.100 |
|  |  |  |  |
| ^a^ Level change refers to an abrupt level change of the Incidence rate between the periods; ^b^ Slope change refers to slope change of the incidence rate over time between the periods. ^c^Time trend refers to the change of Incidence rate associated with a time unit increase. PC= pre-COVID19 phase; SC= School closure phase; MM=Mitigation measures phase. Abbreviations: HRR= Hospitalization Rate Ratio; 95%CI: 95% confidence interval. Note: MM vs. SC contrast was manually added for interpretative purposes without p-value adjustment for multiple comparison. | | | |

| Supplementary Table S10: Interrupted time series analysis results on hospitalisations for Respiratory Diseases and Mental Disorders categories, excluding 0-1years old subjects (sensitivity analysis). | | | |
| --- | --- | --- | --- |
| **Variable** | **HRR** | **95%CI** | **p-Value** |
|  |  |  |  |
| **Respiratory Diseases:** |  |  |  |
|  |  |  |  |
| *Level change^a^* |  |  |  |
| SC vs. PC | 0.15 | 0.08-0.29 | <0.001 |
| MM vs. PC | 0.31 | 0.22-0.43 | <0.001 |
| MM vs. SC | 2.03 | 1.01-4.08 | 0.047 |
|  |  |  |  |
| *Slope change^b^* |  |  |  |
| SC vs. PC | 1.21 | 1.06-1.38 | 0.005 |
| MM vs. PC | 1.05 | 1.02-1.07 | 0.001 |
| MM vs. SC | 0.87 | 0.76-0.99 | 0.037 |
|  |  |  |  |
| *Time trend^c^* | 1.00 | 0.99-1.01 | 0.294 |
|  |  |  |  |
| *Season* |  |  |  |
| Summer | 1.00 |  |  |
| Winter | 1.52 | 1.29-1.81 | <0.001 |
| Spring | 1.30 | 1.09-1.55 | 0.003 |
| Autumn | 1.56 | 1.32-1.84 | <0.001 |
|  |  |  |  |
| **Mental Disorders:** |  |  |  |
|  |  |  |  |
| *Level change^a^* |  |  |  |
| SC vs. PC | 0.49 | 0.29-0.85 | 0.010 |
| MM vs. PC | 1.37 | 1.03-1.81 | 0.030 |
| MM vs. SC | 2.77 | 1.60-4.79 | <0.001 |
|  |  |  |  |
| *Slope change^b^* |  |  |  |
| SC vs. PC | 1.10 | 0.98-1.23 | 0.096 |
| MM vs. PC | 1.01 | 0.99-1.03 | 0.611 |
| MM vs. SC | 0.92 | 0.82-1.02 | 0.120 |
|  |  |  |  |
| *Time trend^c^* | 1.00 | 0.99-1.01 | 0.482 |
|  |  |  |  |
| *Season* |  |  |  |
| Summer | 1.00 |  |  |
| Winter | 0.99 | 0.83-1.18 | 0.938 |
| Spring | 1.14 | 0.95-1.35 | 0.156 |
| Autumn | 1.09 | 0.91-1.29 | 0.349 |
|  |  |  |  |
| ^a^ Level change refers to an abrupt level change of the Incidence rate between the periods; ^b^ Slope change refers to slope change of the incidence rate over time between the periods. ^c^Time trend refers to the change of Incidence rate associated with a time unit increase. PC= pre-COVID19 phase; SC= School closure phase; MM=Mitigation measures phase. Abbreviations: HRR= Hospitalization Rate Ratio; 95%CI: 95% confidence interval. Note: MM vs. SC contrasts were manually added for interpretative purposes without p-value adjustment for multiple comparison. | | | |
