# Supplementary material for: Effects of COVID-19-targeted non-pharmaceutical interventions on pediatric hospital admissions in North Italian hospitals, 2017 to 2022: a quasi-experimental study interrupted time-series analysis
Source: Front Public Health. 2024 Apr 18;12:1393677. doi: 10.3389/fpubh.2024.1393677 (PMC11064846; doi:10.3389/fpubh.2024.1393677)
Supplement: Supplementary file 2 [file Data_Sheet_1.pdf]

Supplementary figure S1: Monthly rate of hospitalization for respiratory diseases, with line trend from ITS regression analysis, in females.

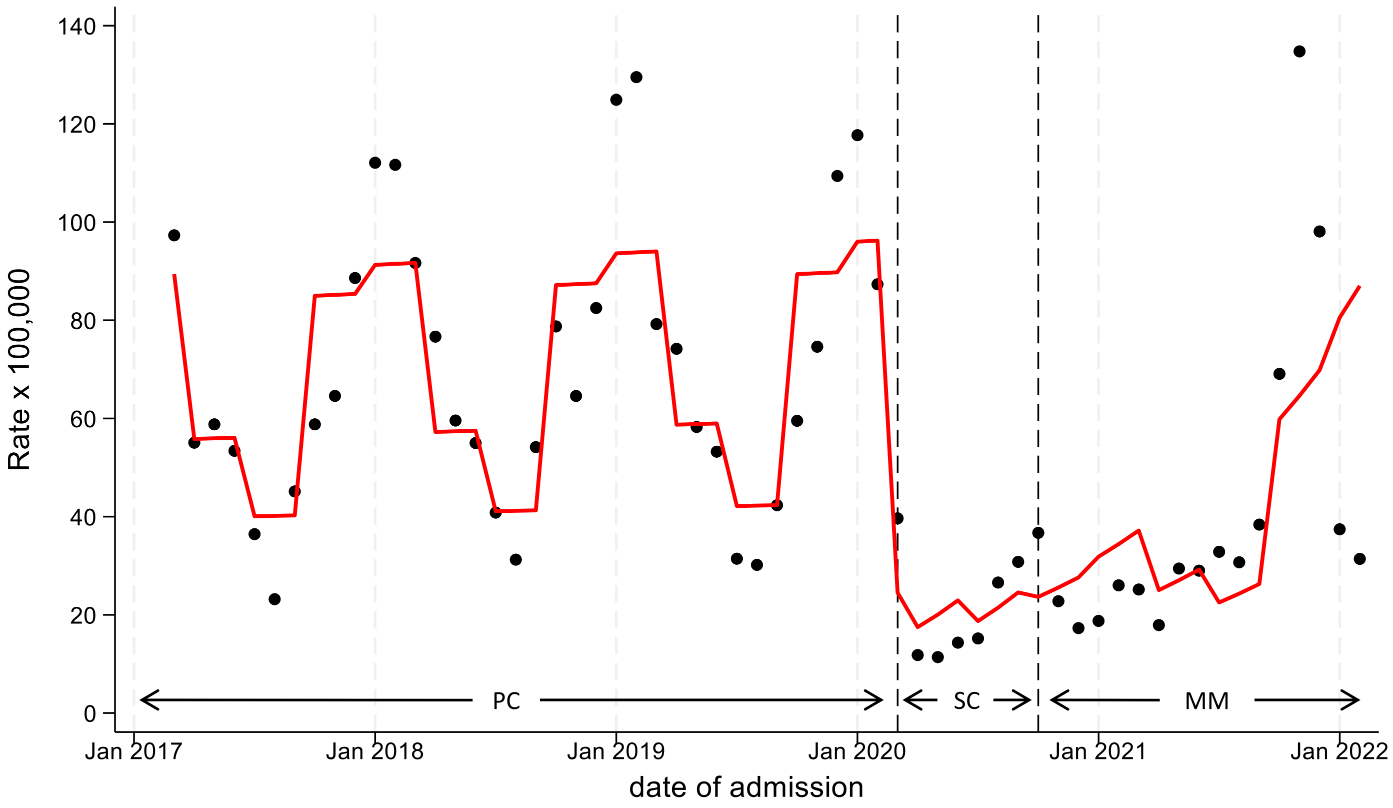

Abbreviations: PC= pre-COVID19 phase; SC= School closure phase; MM=Mitigation measures phase.

Supplementary figure S2: Monthly rate of hospitalization for mental disorders with line trend from ITS regression analysis, in females.

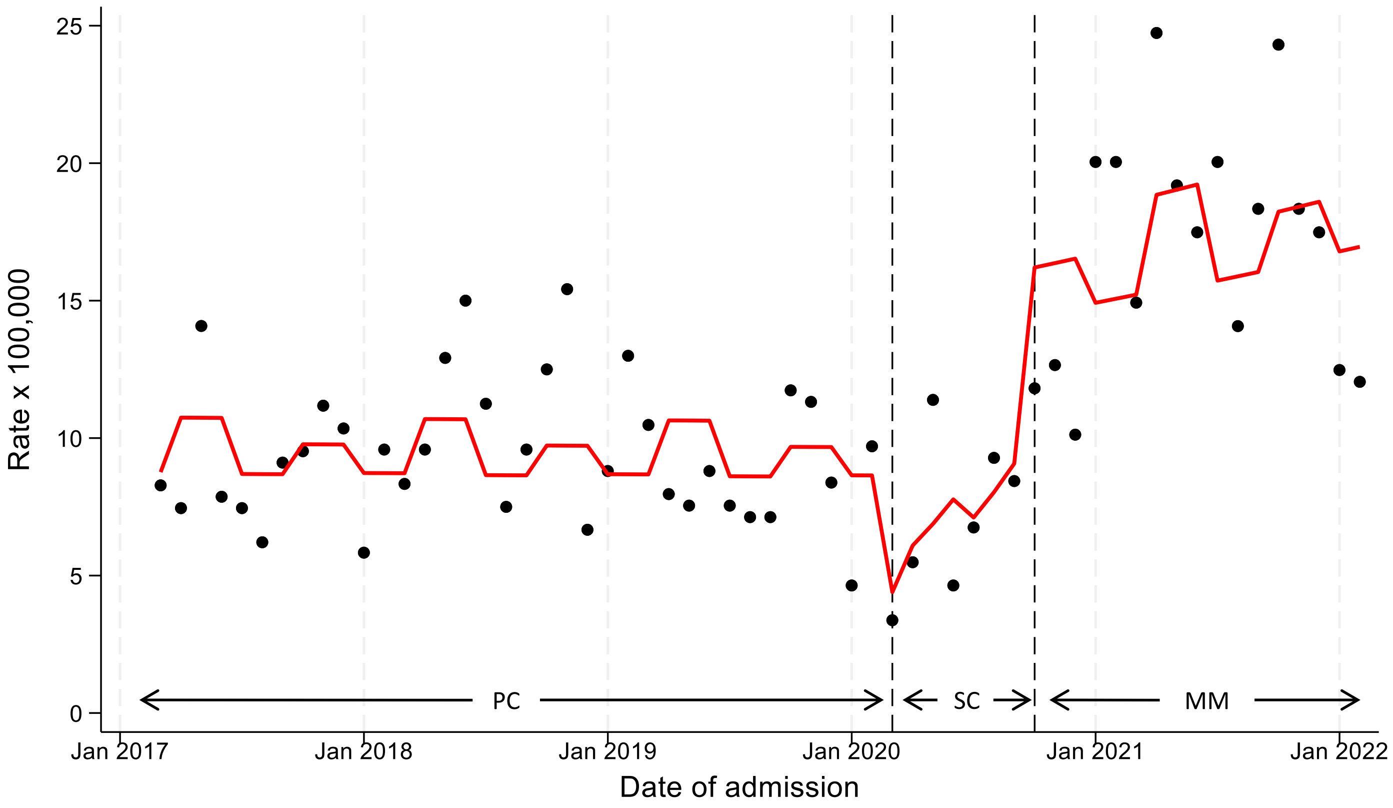

Abbreviations: PC= pre-COVID19 phase; SC= School closure phase; MM=Mitigation measures phase.

Supplementary figure S3: Monthly rate of hospitalization for respiratory diseases, with line trend from ITS regression analysis, in children aged 0-5y.

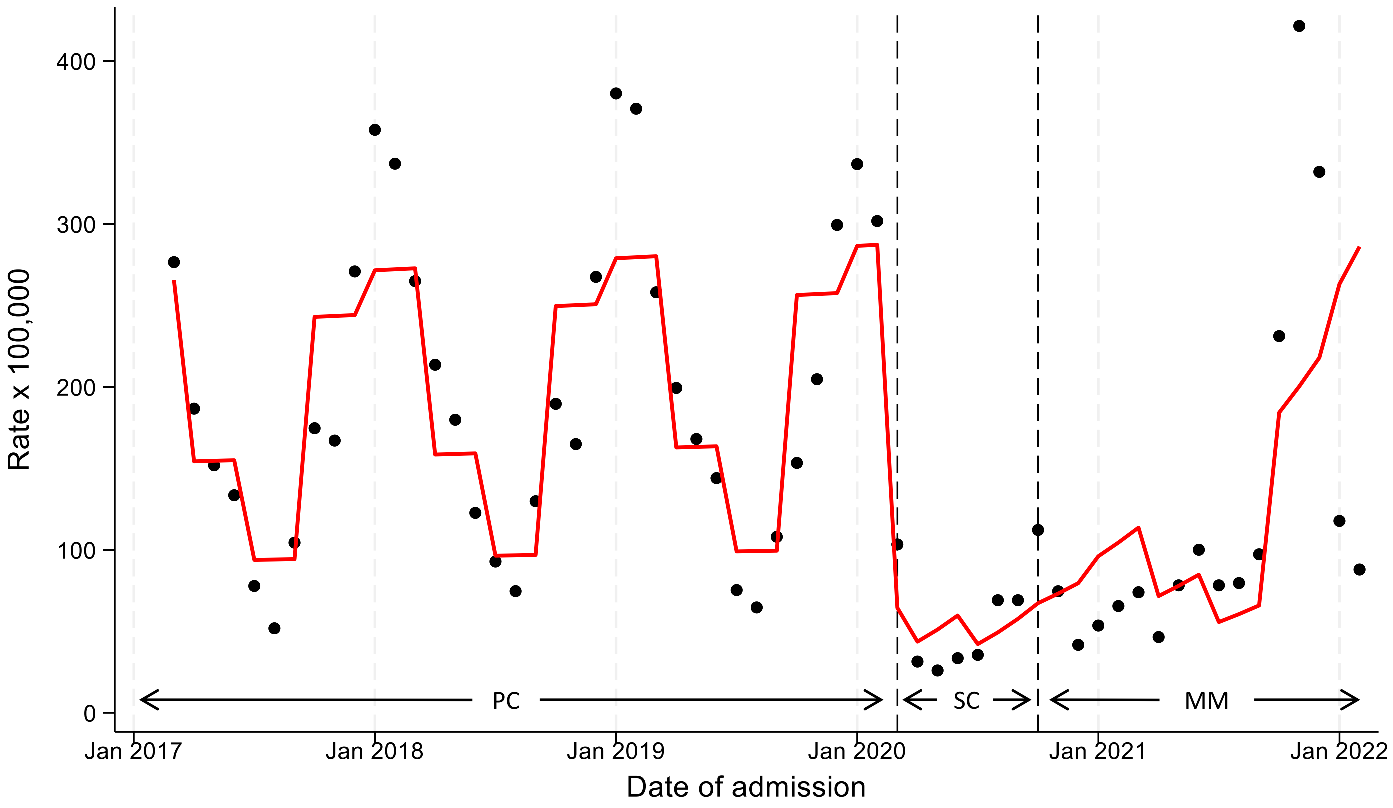

Abbreviations: PC= pre-COVID19 phase; SC= School closure phase; MM=Mitigation measures phase.

Supplementary figure S4: Monthly rate of hospitalization for respiratory diseases, with line trend from ITS regression analysis, in adolescents (age 12-17).

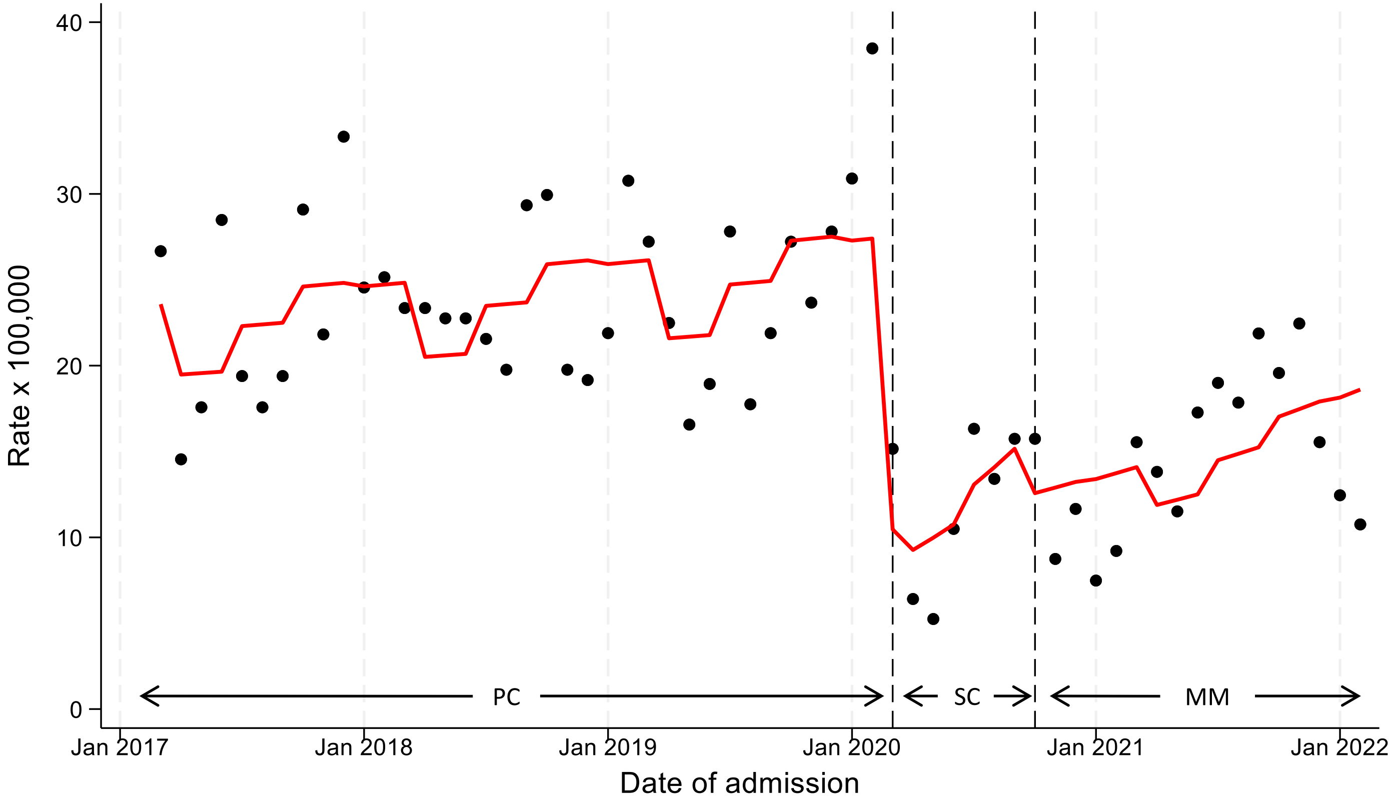

Abbreviations: PC= pre-COVID19 phase; SC= School closure phase; MM=Mitigation measures phase.

Supplementary figure S5: Monthly rate of hospitalization for mental disorders with line trend from ITS regression analysis, in adolescents (age 12-17).

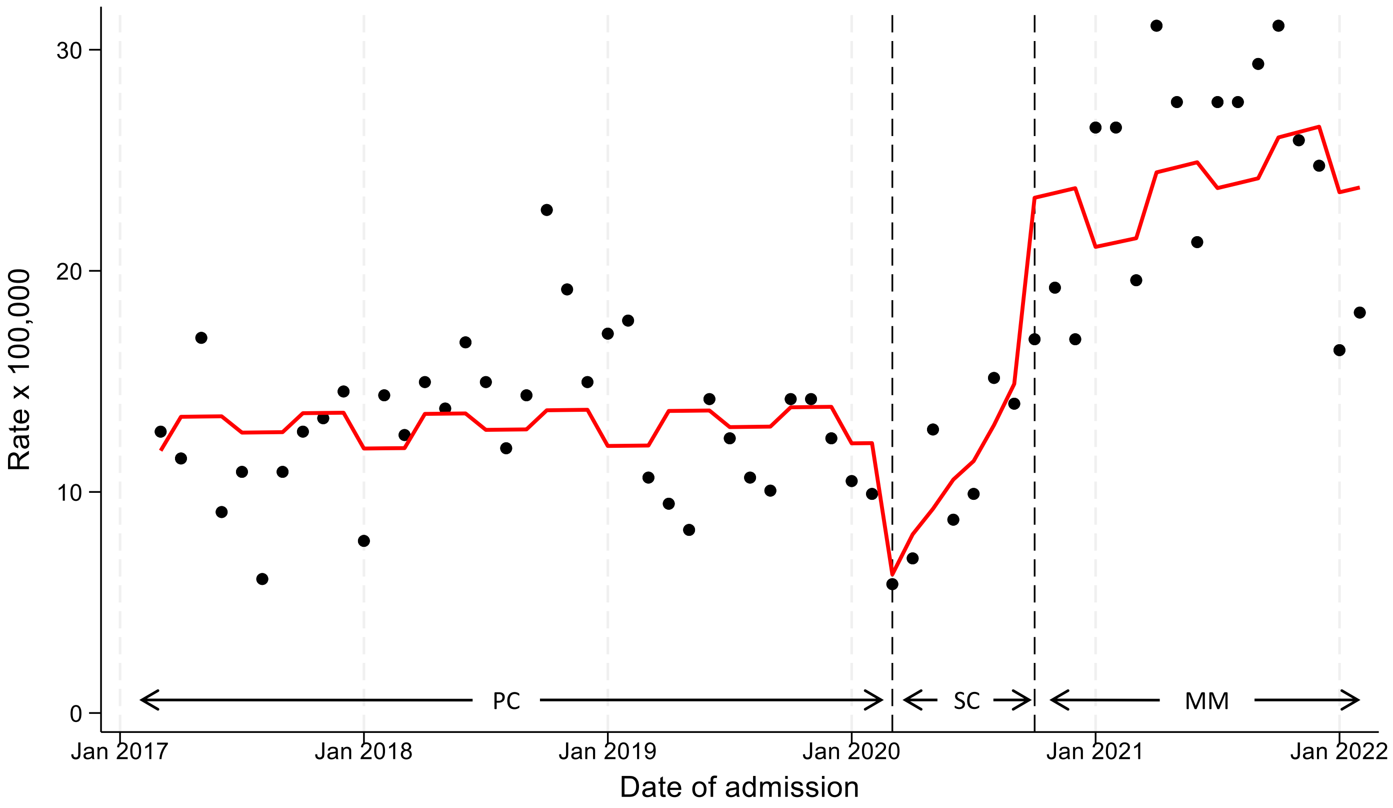

Abbreviations: PC= pre-COVID19 phase; SC= School closure phase; MM=Mitigation measures phase.
